# Adaptions of Lichen Microbiota Functioning Under Persistent Exposure to Arsenic Contamination

**DOI:** 10.3389/fmicb.2018.02959

**Published:** 2018-11-30

**Authors:** Tomislav Cernava, Qerimane Vasfiu, Armin Erlacher, Ines Aline Aschenbrenner, Kevin Francesconi, Martin Grube, Gabriele Berg

**Affiliations:** ^1^Institute of Environmental Biotechnology, Graz University of Technology, Graz, Austria; ^2^Institute of Chemistry, NAWI Graz, University of Graz, Graz, Austria; ^3^Institute of Biology, University of Graz, Graz, Austria

**Keywords:** arsenic pollution, lichen, lichen microbiome, arsenic resistance, holobiont

## Abstract

Host-associated microbiota play an important role in the health and persistence of more complex organisms. In this study, metagenomic analyses were used to reveal microbial community adaptations in three lichen samples as a response to different arsenic concentrations at the sampling sites. Elevated arsenic concentrations at a former mining site expanded the spectrum and number of relevant functions in the lichen-associated microorganisms. Apparent changes affected the abundance of numerous detoxification-related genes, they were substantially enhanced in arsenic-polluted samples. Complementary quantifications of the arsenite S-adenosylmethionine methyltransferase (*arsM*) gene showed that its abundance is not strictly responding to the environmental arsenic concentrations. The analyzed samples contained rather low numbers of the *arsM* gene with a maximum of 202 gene copies μl^-1^ in total community DNA extracts. In addition, bacterial isolates were screened for the presence of *arsM*. Positive isolates were exposed to different As(III) and As(V) concentrations and tolerated up to 30 mM inorganic arsenic in fluid media, while no substantial biotransformations were observed. Obtained data deepens our understanding related to adaptions of whole microbial communities to adverse environmental conditions. Moreover, this study provides the first evidence that the integrity of bacteria in the lichen holobiont is maintained by acquisition of specific resistances.

## Introduction

Lichens are traditional model organisms for symbioses and well-known for their persistence under extreme conditions. In their desiccated stage, they can even survive under Martian conditions simulated on Earth, or even in space (de Vera et al., 2010; Brandt et al., 2015, 2016). Despite their often unnoticed appearance, lichens are found in most terrestrial habitats and cover a substantial portion of Earth’s land surface (Ahmadjian, 1995). In recent years, various molecular and visualizing techniques revealed a highly diversified microbiota colonizing primarily the outer surfaces of lichen symbioses (Cardinale et al., 2008; Grube et al., 2009; Schneider et al., 2011). Owing to the lack of a cuticula in lichens, they are more likely than plants to interact with their surface-colonizing partners. Previously identified functions of the bacterial microbiome include a variety of defense mechanisms that confer enhanced resistance under unfavorable environmental conditions (Cernava et al., 2015; Grube et al., 2015). Moreover, various lichen-associated bacteria can provide essential nutrients to the symbiosis (Sigurbjörnsdóttir et al., 2015). These and various other findings related to the composition and functioning of the lichen microbiota contribute to the accumulating evidence that bacteria other than cyanobionts are an essential part of the symbiosis (Hodkinson and Lutzoni, 2009; Cardinale et al., 2012; Hodkinson et al., 2012; Aschenbrenner et al., 2016; Eymann et al., 2017). The identified ancillary mechanisms provided by highly abundant and diversified microorganisms contribute to the persistence and longevity of the lichen symbiosis (Grube et al., 2015). It was also shown that specialized bacteria can be still detected after several decades on dry-stored lichen samples (Cernava et al., 2016). Considering lichens as microbial multi-species symbioses, also including additional fungi (Spribille et al., 2016), provides the basis for understanding how highly complex functional networks persist under recurrently changing climatic and sometimes hostile conditions. In one of our recent works, we divided beneficial groups of lichen-colonizing microorganisms in two general groups, the “feeders” and the “protectors” (Cernava et al., 2017). The role of the “feeders” fraction is more obvious and often easier to access with various experiment setups. However, both groups are potentially equally important for the persistence of the long-lived symbiosis. The underlying hypothesis of this study was that the holobiont as a whole adapts to environmental stresses. This also includes lichen-associated bacteria; they were subjected to cultivation-dependent and -independent analyses in order to assess functional adaptions to abiotic stress. Their persistence might increase the survivability of the lichen holobiont under unfavorable conditions.

While earlier studies of the lichen-associated microbiome provided a first evidence for the involvement in the detoxification of inorganic substances (e.g., As, Cu, and Zn), the detailed mechanisms remained unknown (Grube et al., 2015). A study by Mrak et al. (2008) demonstrated that lichens can methylate inorganic arsenic, but it remained unknown which symbiosis partner contributed to the observed effects. Arsenicals are widespread in nature and most organisms that were investigated so far have developed resistance mechanisms to counteract toxic effects (Yang and Rosen, 2016). Therefore the species-rich lichen holobiont was expected to harbor various genes in the arsenic resistance (*ars*) operon, including such that are involved in conversions of organic arsenicals. Prevalent genes relevant for biotransformation pathways of As are *arsC* (arsenate reductase), *arsH* (methylarsenite oxidase), *arsI* (C-As bond lyase) and *arsM* [As(III) S-adenosylmethionine methyltransferase; summarized in Yang and Rosen, 2016]. They encode for enzymes that either reduce the toxicity of the arsenicals or that facilitate their exclusion from cells. Other important genes in the *ars* operon that are not linked to transformations of arsenicals are *acr3* (arsenite efflux permease; Wysocki et al., 1997), *arsA* [As(III)-stimulated ATPase; Rosen et al., 1990], *arsD* [As(III) metallochaperone with weak repressor activity; Chen and Rosen, 1997], and *arsR* [As(III)-responsive repressor; San Francisco et al., 1990]. In other studies, plant-associated bacteria in the rhizosphere were shown to methylate arsenic before it is transferred to the host plant (Jia et al., 2013). Such processes represent efficient detoxification cascades where gradual methylation finally volatilizes the As and facilitates its removal from the local environment. Arsenic pollution is widespread in South and East Asia, but also occurs locally in Europe (Ravenscroft et al., 2009), particularly at mining sites. As an example, Gasen Straßegg (Styria/Austria) is a medieval mining site with elevated arsenic concentrations (Geiszinger et al., 2002). This location offers optimal conditions to study the effect of arsenic pollution on the local biota. We selected *Cladonia furcata* (Huds.) Schrad. as the representative model lichen from this sampling site and included four additional lichen species for As and marker gene quantifications. In order to compare functioning of lichen-associated bacteria under different As concentrations, we obtained samples from two additional sampling sites. *Peltigera polydactylon* (Neck.) Hoffm was selected as a representative model from a site with intermediate contamination and *Lobaria pulmonaria* (L.) Hoffm. as a model from a pristine environment. The samples were subjected to a multi-phasic approach combining metagenomics, bioinformatics, analytics, and cultivation studies. Moreover, we implemented a specifically adapted bioinformatics workflow to investigate the spectrum of relevant functions in the respective metagenomes. The resulting findings were discussed in terms of the stability of the lichen-associated microbial community under changing abiotic conditions. They extend our understanding of a highly interesting lifeform and show how the bacterial fraction of the holobiont maintains its integrity despite of unfavorable environmental influences.

## Materials and Methods

### Sampling and Preparation of DNA Extracts for Next-Generation Sequencing

Lichens were sampled from rich populations at an abandoned mining site with elevated arsenic concentrations (Gasen, Austria; 47°23′04.1″N 15°34′30.1″E) and in the proximate vicinity of a city (Graz, Austria; 47°06′45.6″N 15°27′55.8″E). Gasen is an ancient arsenic and gold mining area, located north and northeast of Straßegg pass (1163 m above sea level), covering an area about 1.2 km long and 300 m wide. All samples were visually inspected to detect and minimize the presence of lichenicolous fungi and other organisms. Composite samples that contained multiple lichens (*n* > 20) were used for shotgun sequencing. Each of the composite samples resulted in one metagenomic dataset. Following the method presented by Grube et al. (2015), a total amount of 78.6 g *C. furcata* (Huds.) Schrad. and 47.0 g *P. polydactylon* (Neck.) Hoffm. thalli were separately shock frozen with liquid nitrogen and immediately ground with mortar and pestle. The samples were homogenized in 360 and 270 ml 0.85% NaCl, respectively. Thereafter, the homogenate was filtered using a 63 μm mesh sieve; larger lichen parts were retained and colonizing bacteria were enriched in the filtrate. The filtrate was centrifuged at 8,000 rpm at 4°C for 20 min and the pellet was re-suspended in multiple 1.5 mL 0.85% NaCl aliquots. After a subsequent centrifugation step at 13,000 rpm at 4°C for 20 min, the supernatant was discarded and the pellets were used for parallel DNA isolations (MoBio PowerSoil^®^ DNA Isolation Kit; Carlsbad, CA, United States). Following the DNA isolation, aliquots containing 5.7 μg (*C. furcata*) and 5.8 μg (*P. polydactylon*) of metagenomic DNA were sent to GATC Biotech (Konstanz, Germany) for Illumina sequencing (HiSeq 2500 paired-end runs). During the sequencing, three samples were pooled on one Illumina lane, which resulted in more than 50 million reads per sample. Metagenomic data from *L. pulmonaria* sampled from a site with low arsenic levels was already available from a previous study (Grube et al., 2015; MG-RAST ID: mgm4530091.3). The newly acquired metagenomes for this study were deposited at MG-RAST (^1^Meyer et al., 2008) under ID: mgm4550999.3 (*C. furcata*) and ID: mgm4551030.3 (*P. polydactylon*).

### Screening for Arsenic-Related Functions in the Metagenomes

MG-RAST^1^ was used for a prescreening of arsenic-related functions in the metagenomes. For the presented bioinformatic workflow, paired, non-assembled reads were employed to better account for the sequence abundance. All GenBank^2^ hits obtained with preset parameters on MG-RAST were utilized for further analyses. Searches in the *Cladonia* metagenome yielded 23,261 hits, for *Lobaria* 14,785 hits, and for *Peltigera* 8,388 hits. For in depth analyses of reads assigned to *Planctomycetacia* and *Pedobacter*, 247,221 and 337,587 reads were extracted, respectively. Subsequently, the filtered DNA sequences were aligned to the NCBI-NR protein reference database (date: 05/2015) using the BLAST-compatible local aligner DIAMOND (default settings; version 0.7.9; Buchfink et al., 2015). The BLASTx results were further processed with MEGAN5 (Huson et al., 2011). Functional assignment was performed using the SEED classification system (Overbeek et al., 2005). According to the default settings in MEGAN5 the abundances of the function-assigned sequences were randomly subsampled 1,000 times for contrasting juxtaposition among the three lichen species.

### Isolation of Lichen-Associated Bacteria

Bacterial cultures were obtained according to the protocol described by Grube et al. (2015). After grinding lichen samples with mortar and pestle, a homogenate was prepared using sterile 0.85% NaCl in a 1:10 (w/v) ratio, together with a lab stomacher (BagMixer; Interscience, St Nom, France). Diluted fractions were plated on Nutrient Agar (NA; Sifin, Berlin, Germany) and R2A (Carl Roth, Karlsruhe, Germany). All plates were incubated at room temperature and single colonies were isolated within a period of 2 weeks. The number of *Cladonia*-associated isolates from the sampling site in Gasen (Austria) was 110. In addition, 388 *L. pulmonaria*-associated isolates from our in-house strain collection (Graz University of Technology, Institute of Environmental Biotechnology) were included and subjected to the same screening approaches. These isolates were obtained from the same sampling location (Johnsbach, Austria) as the material for metagenome sequencing of *L. pulmonaria*.

### PCR-Based Screening for the arsM Gene Within Cultivated Bacteria

A total of 498 isolates was used for DNA extraction and subsequent PCR amplifications with the primer pair arsMF1/arsMR2 (Jia et al., 2013). The results of the amplifications were controlled on 0.8% agarose gels. Bands that correspond to the expected size of the arsMF1/arsMR2 product (346 bp for *Rhodopseudomonas palustris* CGA009) were excised from the gels, purified with the Wizard SV and PCR Clean-Up System (Promega, Madison, WI, United States), and sent for Sanger sequencing (LGC Genomics, Berlin, Germany). BLASTn searches against the NCBI^3^ nucleotide database were used to evaluate the identity of the sequences.

### Cultivation of Bacteria in Arsenic-Supplemented Media

Nutrient Broth (NB; Sifin, Berlin, Germany) was supplemented with arsenate (Na_2_HAsO_4_ × 7 H_2_O) and arsenite (NaAsO_2_; Merck, Darmstadt, Germany) to test the resistance of *arsM* carriers toward inorganic arsenic. Bacterial isolates were grown in over-night cultures and subsequently transferred to 20 ml flasks with 0 – 20 mM As(III) and 0 – 30 mM As(V). The growth was monitored over a period of 28 h by spectrophotometric analysis and the determination of OD_600_ values.

### Extraction of Arsenic From Bacterial Samples

Standards and instrumentation for the identification and quantification of arsenic species in lichen samples are described in the Supplementary Data file. Approximately 20 mg of each bacteria sample and reference material ERM – BC 211 (Rice) were weighted into Eppendorf vials (1.5 mL). In each of them 1 mL of water was added as an extraction solution. Each aqueous suspension was homogenized by sonication for 30 s. This process was repeated 6 times with cooling intervals of 2–3 s between each sonication by using liquid nitrogen. Liquid nitrogen was used for a faster cooling process of the hot sonicated samples and to enhance the disruption of the cells by fast temperature changes. The extracts were centrifuged for 15 min at 4700 rpm. Each supernatant was filtered through a 0.2 μm membrane filter and placed in 0.6 mL polyethylene vials. These supernatants were used directly for HPLC-ICPMS analysis. After the first extraction, the pellets of each bacterial isolate and reference materials were washed three times with water and digested in HNO_3_ using the microwave digestion system. Digested samples were then analyzed by ICPMS, using the measurement conditions described below, to determine the total arsenic content of the pellets.

### Total Arsenic Determination

Approximately 20 mg of the samples and reference materials were weighed into quartz tubes (12 mL). In each of them 2 mL water and 2 mL HNO_3_ was added for digestion using the microwave digestion system. The digested solutions were allowed to cool to room temperature, transferred to polypropylene tubes (15 mL) and diluted with water to 10 mL. To all digested samples and calibration standards an internal standard solution containing Ge and In was added giving a final concentration of 10 μg /L of each of them. Calibration standards were prepared in 20% HNO_3_ for matrix matching with the digested samples. ICPMS measurements were performed in the collision cell mode (He, 5 mL/min) to minimize polyatomic interferences from argon chloride (^40^Ar^35^Cl) on arsenic (^75^As). With this method for total As measurements, the certified reference material ERM – BC 211 (Rice, certified [As] = 260 ± 13 μg/kg) returned a value of 284 ± 36 μg/kg, *n* = 5); San Joaquin soil NIST SRM 2709 (certified [As] = 17.7 ± 0.8 mg/kg) returned a value of 16.8 ± 1.2 mg/kg, *n* = 4; and lichen IAEA 336 (certified [As] = 0.55 – 0.71 mg/kg, 95% confidence limits) returned a value of 0.74 ± 0.06, *n* = 4. Measured values are mean ± 2SD.

### Identification and Quantification of Arsenic Species in Bacterial Samples

For arsenic speciation measurements, the Agilent 1100 series HPLC connected to an Agilent 7900 ICPMS was used. Anion-exchange HPLC was performed using a PRP X100 column (150 × 4.6 mm, 5 μm particle size, Hamilton Company, Reno, NV, United States) and a mobile phase of malonic acid 10 mM pH 5.6 (adjusted using aqueous ammonia; column temperature was 40°C, flow rate 1 mL/min and injection volume was 20 μL. Cation-exchange HPLC was performed with an IonoSpher C column (3.0 × 100 mm, 5 μm particle size, Agilent Technologies, Germany) with a mobile phase of 10 mmol⋅L^-1^ pyridine buffer adjusted to pH 2.6 with formic acid at a flow rate of 1 mL min^-1^. Column temperature was 40°C and the injected sample volume was 20 μL. For both anion- and cation-exchange HPLC, the monitored ICPMS signals were m/z 75 (^75^As and ^40^Ar^35^Cl) and m/z 77 (^40^Ar^37^Cl, to assess possible chloride interferences), and for internal standards m/z 74 (^74^Ge) and m/z 125 (^125^Te). For ICPMS measurements, a carrier gas (Ar) and an optional gas (5% CO_2_ in argon to enhance arsenic response) were used. For identification and quantification of As compounds in the extracts, respective chromatographic peak areas were compared with eight As compounds used as standards [As (III), As(V), DMA, MA, AB, TMAO, AC, Tetra]. However, quantifiable peaks were obtained only for As (III), As(V), and DMA. The presence of As(III) was also assessed by repeated HPLC analysis of the sample after addition of H_2_O_2_, and observing the removal of the peak for As(III) and a concomitant increase in the As(V) peak (Scheer et al., 2012).

### qPCR-Based Quantification of Bacteria and arsM

Quantifications were done with total DNA extracts from lichen samples obtained from three sampling sites with varying As concentrations. *Pseudovernia, Platismatia, Usnea, Hypogymnia*, and *Cladonia* samples were collected at a site with high As prevalence (Gasen, Austria; 47°23′04.1″N 15°34′30.1″E). *Peltigera* samples were obtained from a site showing intermediate As prevalence (Graz, Austria; 47°06′45.6″N 15°27′55.8″E), while *Lobaria* was sampled at lowest environmental As concentrations (Johnsbach, Austria; 47°32′35″N 14°37′38″E). Quantification of *arsM* genes in the lichen DNA extract was conducted with primer pair arsMF1/arsMR2, as described by Jia et al. (2013). Standards containing the *arsM* fragments were prepared with total DNA extracts from lichen samples. Briefly, the gene fragments from the total community DNA of a *L. pulmonaria* sample were cloned into the pGEM^®^-T Easy Vector (Promega, Madison, WI, United States) and later re-amplified with vector specific primers. The integrity of the fragment was confirmed by Sanger sequencing and subsequent alignment within the NCBI database. Amplification-grade DNase I (Sigma-Aldrich, St. Louis, MO, United States) treated total DNA extract was used to determine inhibitory effects of co-extracted substances. Based on this experiment, the total community DNA was diluted 1:10 and target genes were amplified using KAPA SYBR FAST qPCR Kit (Kapa Biosystems, Woburn, MA, United States). Two independent runs, with three replicates for each sample, were performed on the Rotor Gene 6000 (Corbett Research, Mortlake, VIC, Australia). For the quantification of bacteria in lichen samples the same protocol was used with primers targeting the 16S rDNA region. Therefore, the primer pair Unibac-II-515f/Unibac-II-927r was utilized as described by Lieber et al. (2003), and standards containing the Unibac-II fragments were prepared according to Köberl et al. (2011). The specificity of the amplicons was assured with both, melting-curve analysis and gel-electrophoresis of the qPCR products, respectively. In addition, sufficient amplification efficiency was confirmed. Gene copy numbers for *arsM* and the Unibac-II fragments were calculated per μl community DNA extract.

## Results

### Identification of Lichen-Associated Bacteria With Arsenic-Related Functions

**FIGURE 1:**
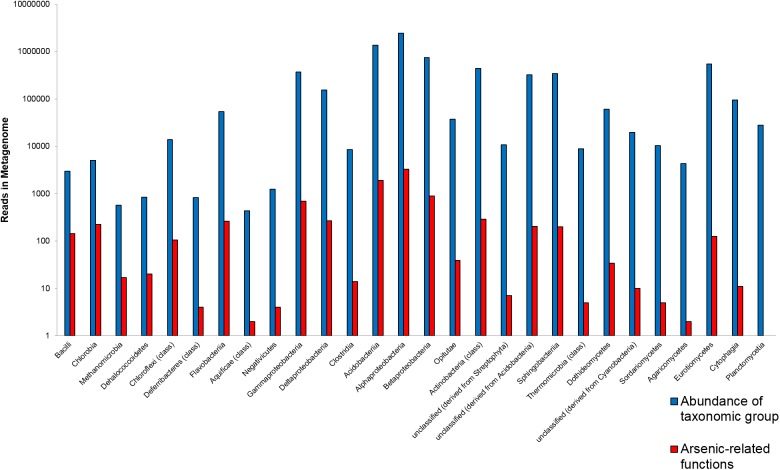
Overview of the most abundant carriers of arsenic-related functions in the *Cladonia* *furcata* metagenome. The abundance of specified taxa (blue bars; class level) is correlated to the number of all assigned functions related to arsenic tolerance and its utilization (red bars). Corresponding data for taxonomic and functional analyses was retrieved from MG-RAST (http://metagenomics.anl.gov; Meyer et al., 2008).

In a first assessment, various lichen-associated microorganisms from the sampling site with elevated arsenic levels (Gasen, Austria; 47°23′04.1″N 15°34′30.1″E) were shown to carry genes coding for arsenic-related functions. The analysis was performed with shotgun sequences of a *C. furcata* metagenome from the above mentioned sampling site. The analysis summarizes gene abundances on class level for a general overview (Figure 1). In total 22 bacterial classes, four fungal classes, one archaeal class and one plant-derived group were identified as carriers of arsenic-related genes. The proportionally highest number of such genes was found in *Bacilli*. Here 4.8% of the assigned bacteria coded for an arsenic-related function. Other taxa with a high proportion of the investigated function were assigned to *Chlorobia* (4.4%), *Methanomicrobia* (Archaea; 3.0%), and *Dehalococcoidetes* (2.4%). The highly abundant *Alphaproteobacteria* showed an occurrence rate of 0.13% for arsenic-related functions. Lichen-associated bacteria with a comparatively lower proportion of arsenic-related functions included *Cyanobacteria* with a 0.05% occurrence rate.

**FIGURE 2:**
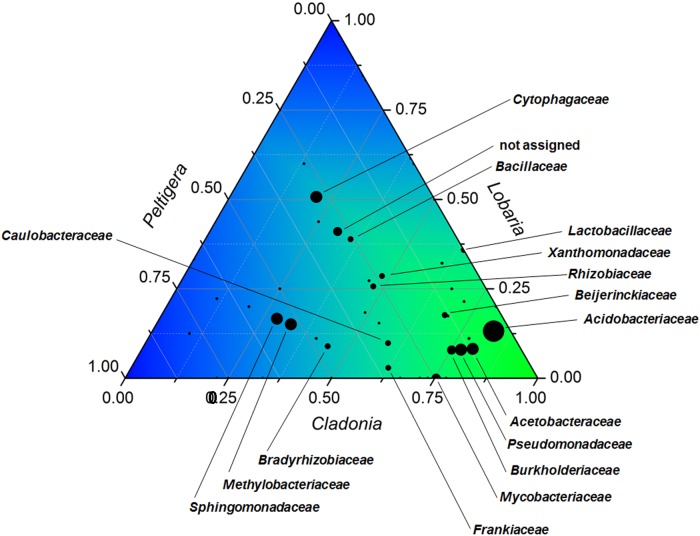
Ternary plot visualizing identified carriers of arsenic-related genes and their occurrence in the three analyzed lichens. The taxonomic composition is shown at family level for bacteria that hold arsenic-related functions in *C. furcata*, *Lobaria pulmonaria*, and *Peltigera polydactylon*. The bubble sizes correspond to the total abundance of each taxon. Taxa with a total occurrence <50 were not annotated. Origin v8.5 (http://originlab.com) was utilized for the visualization of the ternary diagram.

When all three analyzed lichen metagenomes were compared, it was shown that most bacteria encoding for arsenic-related functions were present in all samples, although in varying proportions (Figure 2). Distinct *Cladonia*-associated bacteria were increased in the analyzed sample with the elevated arsenic content. The most apparent increase in abundance for prevailing taxa (more than 300 reads) was observed for the families *Acetobacteraceae* (9.8-fold increase compared to *L. pulmonaria*), and *Acidobacteriaceae* (6.3-fold increase). *Mycobacteriaceae* with genes encoding for arsenic-related functions were only found in the *Cladonia* and *Peltigera* metagenomes with 95 and 31 hits, respectively.

### Assessment of Arsenic-Related Functions in the Lichen Microbiome

**FIGURE 3:**
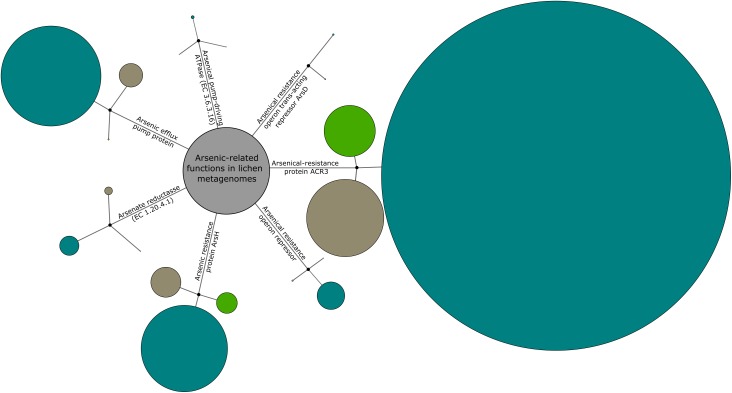
Overview of the prevailing arsenic-related functions in the metagenomes. *C. furcata* (blue circles) samples were obtained in a locally constrained environment with high As concentrations (Gasen/Austria). *P. polydactylon* (green circles) was obtained from close vicinity to an urban area (Graz/Austria). The third sample, *L. pulmonaria*, (brown circles) was collected at a site with the lowest As concentrations (Johnsbach/Austria). The circle sizes correspond to the normalized number of metagenome reads for each function. Additional information on As concentrations at the sampling sites is presented in Supplementary Table S1.

BLASTx analysis of the pre-filtered metagenomic sequences obtained from three different lichen-associated microbiomes yielded 13,887 sequences assigned to arsenic-related functions. The majority of these sequences (73%; 10,138/13,887) originated from the *Cladonia*-associated microbiome (Figure 3). In comparison, the *Lobaria-* and *Peltigera*-associated microbiomes contributed approximately 18% (2,450/13,887) and 9% (1,299/13,887) to the total of detected arsenic-related sequences. The arsenical-resistance protein ACR3, which belongs to the family of arsenite (As^3+^) permeases, was found to be the most abundant protein in total (59.3% of all function-related sequences). Other abundant proteins were the arsenic efflux pump (15.6%; 2165/13,887) and the arsenic resistance protein ArsH with methylarsenite oxidase activity (17.1%; 2376/13,887) – both involved in arsenic resistance mechanisms. However, other detected enzymes, which are involved in arsenic-related functions, were found to be less abundant. One of those enzymes was the arsenate reductase (EC 1.20.4.1; 3.54%; 492/13,887), which is part of an arsenate detoxification system yielding arsenite. Although arsenite is a more toxic form of arsenic, it can be extruded by the ion transport machinery arsenite-transporting ATPase, which could be also detected within the investigated lichen-associated metagenomes (arsenical pump-driving ATPase; EC 3.6.3.16; 0.53%; 73/13,887). The least abundant function-related sequences were assigned to two different kinds of arsenic metabolism-related repressors including the arsenical resistance operon repressor (ArsR; 3.7%; 511/13,887) as well as the weak arsenical resistance operon repressor with metallochaperone activity ArsD (0.21%; 29/13,887). The latter one could not be detected in the *Lobaria*-associated microbiome at all. Two bacterial lineages from the *Cladonia*-associated microbiome were subjected to deepening analyses in order to screen for As-related genes. Taxon-specific reads were extracted for *Planctomycetacia* which had no As-related assignments in the automated analysis and for *Pedobacter* as a representative for an As-tolerant isolate. SEED-based functional profiles were constructed for both lineages (Supplementary Figures S1, S2). Within *Planctomycetacia*-assigned reads, one hit for arsenate reductase (EC 1.20.4.1) and one hit for arsenical-resistance protein ACR3 was detected. For *Pedobacter*-assigned reads, eight distinct hits for arsenical-resistance protein ACR3 were detected.

### Quantification of Arsenic Levels in Lichens and Bacterial Cultures

The quantification of arsenic in lichen samples from three distinct locations showed elevated levels of arsenic in those samples collected in the contaminated region (Supplementary Table S1). The arsenic content ranged from 0.72 mg/kg (*Usnea*) to 6.4 mg/kg (*Pseudovernia*). The *Cladonia* sample, which was utilized for metagenome sequencing, had an arsenic content of 2.3 mg/kg. The other two lichens utilized for the metagenome comparisons – *Lobaria* and *Peltigera* – were collected from outside the contaminated site and had arsenic contents of 0.27 and 1.0 mg/kg, respectively. The selected lichen models can only grow on certain substrate types, therefore respective substrate samples were also collected and analyzed (Supplementary Table S1). Here, the soil sample obtained close to the spot where *Cladonia* was sampled, had an exceptional arsenic content of 72.9 mg/kg soil, while the arsenic concentration close to the *Peltigera* sample was determined to be 7.2 mg/kg. The soil in close proximity to the *Lobaria* sample had an arsenic content of 4.4 mg/kg, while the tree bark, which serves as an anchor for its epiphytic lifestyle had only 0.02 mg/kg total arsenic content. The removal of the outer lichen layer with sodium hypochlorite resulted in increased arsenic contents with the exception of an *Lobaria* sample (Supplementary Table S1). The lichen samples were visually inspected after the treatments to assure that deeper layers were not affected; however, the extracellular matrix might have been compromised during the exposure to NaClO. Overall, an approximately twofold increase in arsenic content was observed for the analyzed lichens. This indicates that inner structures of the lichen accumulate higher arsenic amounts than the bacteria-rich outer layers.

Bacterial isolates, which were identified as potential biotransformers of arsenic due to the presence of the *arsM* gene, were assigned to the genera *Leifsonia, Micrococcus, Pedobacter, Staphylococcus*, and *Streptomycetes*. These isolates were isolated from the same lichen samples that were used for the metagenome analyses. They were cultivated in As(III)- and As(V)-supplemented media to determine potential formation of methylated arsenic species. Subsequently, the bacterial cells were freeze-dried and subjected to inductively coupled-plasma mass-spectrometry (ICPMS) analyses. Global arsenic quantification and speciation showed that cultivation of the microorganisms in arsenic-rich media was followed by accumulation of up to 9.8 mg/kg As(III) in *Staphylococcus warneri* 50P3R (Table 1). *S. warneri* 50P3R was the only isolate that tolerated elevated As(III) levels (>5 mM) in the cultivation media (Supplementary Figure S3). In contrast, four lichen-associated bacterial isolates from the combined culture collections tolerated As(V) concentrations of 30 mM (strains 29P4R, 50P3R, 77P3BRAB, and 583P1B; Supplementary Figure S3). The cultivation of the isolates was always followed by an enrichment of inorganic arsenic in the bacteria (Table 1). Here, *S. warneri* 50P3R was again the isolate with the highest accumulation. The analyzed cell dry weight contained 7.30 mg/kg As(V). However, neither significant shifts of the As(III)/As(V) ratio, nor the occurrence of additional arsenic species in bacteria and cultivation media was observed. The occurrence of additional arsenic species was primarily triggered by the increase of inorganic arsenic in the cultivation media (Table 2). Due to their relatively small cell mass, bacterial cultivation in As-supplemented media was not accompanied by a significant decrease in the total arsenic content of the medium.

**Table 1:** Concentrations (μg As/kg dry mass) of total arsenic (by ICPMS) and of arsenic species (by HPLC/ICPMS) in bacterial cells following cultivation in arsenate solutions.

Arsenic exposure level in nutrient broth used for cultivation	Isolate	Total As (μg/kg)	Extraction efficiency (%)	iAs^3+^ (μg/kg)	DMA (μg/kg)	iAs^5+^ (μg/kg)	Sum of species^#^	Column recovery (%)
–	*Micrococcus luteus* 29P4R	27 ± 3	56	3 ± 1	4 ± 1	3 ± 2	10	67
	*Leifsonia poae* 583P1B	41 ± 5	76	10 ± 5	9 ± 4	9 ± 4	28	90
	*Staphylococcus warneri* 50P3R	110 ± 10	21	5 ± 2	2 ± 1	7 ± 3	14	61
0.01 mM As^5+^	*Micrococcus luteus* 29P4R	1883 ± 180	20	301 ± 30	n.q	26 ± 5	327	89
	*Micrococcus luteus* 29P4R^∗^	–	–	≤0.1	6 ± 2	482 ± 40	488	–
	*Staphylococcus warneri* 50P3R	1696 ± 20	6	78 ± 7	n.q	15 ± 3	93	89
	*Staphylococcus warneri* 50P3R^∗^	–	–	≤0.1	3 ± 1	143 ± 10	146	–
3 mM As^5+^	*Micrococcus luteus* 29P4R	4008 ± 270	46	1484 ± 60	n.q	152 ± 10	1636	88
	*Micrococcus luteus* 29P4R^∗^	–	–	≤0.1	16 ± 6	2051 ± 720	2067	–
	*Micrococcus luteus* 77P3BRAB	5439 ± 300	47	1610 ± 50	n.q	550 ± 90	2160	85
	*Micrococcus luteus* 77P3BRAB^∗^	–	–	≤0.1	21 ± 7	3353 ± 160	3374	–
	*Leifsonia poae* 583P1B	3147 ± 450	63	1503 ± 190	n.q	285 ± 80	1788	90
	*Leifsonia poae* 583P1B^∗^	–	–	≤0.1	10 ± 3	2393 ± 330	2403	–
	*Staphylococcus warneri* 50P3R	7300 ± 1000	32	1421 ± 120	n.q	662 ± 210	2083	90
3 mM As^3+^	*Staphylococcus warneri* 50P3R	9764 ± 980	38	2771 ± 80	n.q	504 ± 110	3275	87


*Values are mean ± standard deviations of analytical results for three HPLC measurements per bacterial isolate. ^∗^Supernatant of bacterial extracts containing 10% H_2_O_2_ [to convert As(III) into As(V)]. ^#^The total number of As-containing compounds in the sample.*

**Table 2:** Arsenic species (μg As/kg) in nutrient broth of distinct bacterial isolates.

	Nutrient broth of corresponding bacteria	Total As (μg/kg)	iAs^3+^ (μg/kg)	DMA (μg/kg)	iAs^5+^ (μg/kg)	Sum of species^#^	Column recovery (%)
Before cultivation	*Micrococcus luteus* 29P4R	1057 ± 90	46 ± 5	1 ± 0.1	610 ± 10	657	94
	*Micrococcus luteus* 29P4R^∗^	–	1 ± 0.1	2 ± 0.3	651 ± 10	654	90
	*Staphylococcus warneri* 50P3R	1069 ± 40	41 ± 4	2 ± 0.2	661 ± 14	704	94
	*Staphylococcus warneri* 50P3R^∗^	–	≤0.1	1 ± 0.1	659 ± 20	660	90
After cultivation	*Micrococcus luteus* 29P4R	920 ± 270	113 ± 10	3 ± 0.3	558 ± 12	674	97
	*Micrococcus luteus* 29P4R ^∗^	–	≤0.1	4 ± 0.4	633 ± 10	637	90
	*Staphylococcus warneri* 50P3R	1009 ± 130	43 ± 4	4 ± 1	619 ± 20	666	93
	*Staphylococcus warneri* 50P3R^∗^	–	trace	4 ± 0.4	618 ± 13	622	88


*The arsenic content of in the nutrient broth was 0.01 mM As(V) (=750 μg As/kg) prior to cultivation. Values are mean ± standard deviations of analytical results for three measurements per sample. ^∗^Nutrient broth containing 10% H_2_O_2_. ^#^The total number of As-containing compounds in the sample.*

### Correlation Between High Arsenic Concentrations in the Local Environment and the *arsM* Gene Copy Number

**FIGURE 4:**
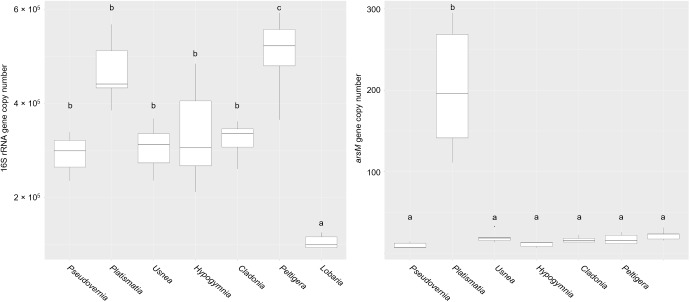
Quantification of the 16S rRNA gene fragment **(A)** and *arsM* **(B)** in lichen samples. Gene-specific primer sets were used for the RT qPCR-based quantifications in lichen DNA extracts. Lichen samples were obtained from three sampling sites with varying As concentrations. The gene copy numbers were calculated per μl total DNA extract. Statistical analysis was employed to identify significant (*p* < 0.05) differences in gene copy numbers. Different letters were used to differentiate between statistically discriminative groups. Additional information on As concentrations at the sampling sites is presented in Supplementary Table S1.

In order to obtain information on the methylation potential of inorganic arsenic by lichen-associated bacteria, the *arsM* gene was quantified by RT qPCR. In total, five lichen samples from the contaminated region and two reference samples (*L. pulmonaria* and *P. polydactylon*) were analyzed. Furthermore, a potential correlation between the number of colonizing bacteria and the occurrence of the *arsM* gene was evaluated. In the context of bacterial counts, *Peltigera* and *Platismatia* demonstrated the highest colonization rates, while *Lobaria* was the lichen with the lowest number of 16S rRNA gene fragments (Figure 4A). Increased *arsM* gene counts were only obtained with the *Platismatia* sample, while the other lichens had insignificant numbers of this gene (Figure 4B). The calculated *arsM* gene copy number for the total DNA extract from *Platismatia* had a mean value of 202 copies μl^-1^. There was no evident correlation between the 16S rRNA gene copy number and the *arsM* gene copy number, indicating that the methylation potential of inorganic arsenic is not enhanced by the colonization density.

## Discussion

**FIGURE 5:**
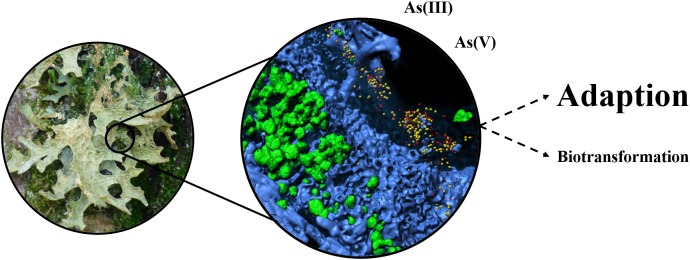
Schematic illustration of the main identified responses of the lichen microbiota to arsenic pollution. The model summarizes the overall findings of the integrative approach. Metagenomic data indicated high frequencies of arsenic resistance mechanisms in the sample from the polluted environment. The implementation of cultivation-dependent analyses provided additional evidence for the adaptability of the lichen microbiota to elevated As concentrations. Biotransformation primarily refers to the methylation of arsenic, which was addressed within the present study.

The present study sheds light on the role of the lichen-associated microbiota under the persistent presence of arsenic in the adjacent environment. In general, the obtained data indicate an adaptation of the present bacteria under arsenic exposure. The analyzed metagenomes provided evidence for the accumulation of arsenic-specific resistance mechanisms when the lichen was located in an arsenic-polluted environment. Such adaptations facilitate persistence of the microbiota and thus might be crucial to preserve symbiosis-relevant functioning (Figure 5). By enduring unfavorable conditions in typical lichen habitats, which primarily include high fluctuations in humidity and temperature, the associated bacteria can fulfill their main roles as (i) protectors against biotic and abiotic stresses, (ii) providers of nutrients, vitamins and co-factors, and (iii) detoxifiers of organic and inorganic compounds (Grube et al., 2015; Cernava et al., 2017; Eymann et al., 2017).

The global assessment of arsenic pollution on the lichen microbiome showed that integral members of the lichen microbiome, i.e., various taxa belonging to *Alphaproteobacteria*, are adaptable to this rather unusual environmental condition. While most of the highly abundant microbial lineages harbored varying abundances of As-related genes, *Planctomycetacia* was the only bacterial class without any hits in this assessment. The extraction of a subset within the *Cladonia* metagenome, that contained all reads assigned to this class, and subsequent BLAST searches resulted in two hits with implications in biotransformation and resistance toward arsenicals. In contrast, the extraction of reads assigned to the genus *Pedobacter*, which was also represented by an As-tolerant isolate, led to the identification of more As-relevant genes. In general, the results obtained with metagenomic analyses indicate that a high diversity is maintained in the lichen microbiome irrespective of the presence of arsenicals in the environment. Previous metagenomics-based analyses support this findings and also showed that As-contamination in the environment is not restrictive for microbial diversity (Suhadolnik et al., 2017). Complex microbial communities can support functional diversity related to As transformation and contribute to the mobility of arsenicals in sediments. In the present study a similar structural diversity of involved microorganisms was identified; however, the composition of the community indicated host-specificity. Lichens represent a widespread lifeform that can thrive on a wide range of substrates, including soils, rocks, and various living as well as dead plants (Nash, 2008). Although these composite organisms are well adapted to various climatic zones, they are often highly sensitive to air pollution (Scheidegger and Werth, 2009). These contrasting characteristics of the lichen symbiosis provided additional ground to investigate the effects of arsenic pollution on the lichen microbiome. More importantly, the microbiome-focused experiments deepen and expand the study presented by Mrak et al. (2008) that focused on arsenic methylation in lichens. In addition, the obtained results help to better understand the distribution of functional roles in the lichen holobiome. Metagenomic analyses showed a disproportional distribution of resistance genes in all three lichen-derived datasets and a prevalence of hits for arsenical-resistance protein ACR3. Although resistance genes occur in specific operons, their composition can vary and only distinct genes are always associated with each other (Yang and Rosen, 2016). The current data implies that bacteria have a rather subsidiary role in the methylation of inorganic arsenic. Nevertheless, the effects of elevated arsenic concentrations in the environment were evident when metagenomic data were compared. Various arsenic-specific detoxification mechanisms were present across previously described members of the lichen holobiont. The evident increase of genes encoding the arsenic resistance proteins ACR3 and ArsH as well as the arsenic efflux pump in the *Cladonia* metagenome provides a major indication for the adaptions of the microbiome toward the present arsenic.

The focus of this study was to elucidate functioning in the lichen microbiome under different arsenic concentrations. Therefore, we selected one representative lichen species from each sampling site and extracted arsenic-specific functions from their metagenomes. The main determinant for including a distinct lichen species in this study, was the presence of a large and healthy lichen population. This was primarily required for total community DNA extractions and subsequent metagenome sequencing. Addressing species-specific changes in the microbiome would require additional experiments including more datasets of one distinct lichen species. For this purpose, amplicon sequencing of the 16S rRNA gene would be more suitable than multiple metagenome analyses considering its lower cost and better efficiency in deciphering community structures. In this context, previous studies identified the geographical location and the photobiont type as important shapers of the microbial community structure (Cardinale et al., 2012; Hodkinson et al., 2012). Moreover, it was also shown that lichens can vertically transfer their microbiome to equip the successive generation (Aschenbrenner et al., 2014). This observation provides further indications for the significance of the microbiome for the symbiotic system. Comparable mechanisms were shown for a broad range of plants that benefit from a highly diversified microbiome (Berg et al., 2014; Hardoim et al., 2015). It remains to be evaluated if arsenic pollution eradicates any complementary functions in the lichen microbiome while sparing those required for survival.

Lichens are remarkably efficient accumulators of inorganic elements. This ability is vital for their commonly epiphytic lifestyle, but can also result in adverse effects in polluted environments (Ahmadjian, 1993). When the surface of lichens was treated with sodium hypochlorite solution, we observed higher concentrations of arsenic in the remaining tissues. This is in accord with previous studies where accumulation of inorganic arsenic in lichen tissues was shown following *in vitro* exposure (Mrak et al., 2010). The accumulation of various metalloids and heavy metals by lichens can be used for biomonitoring purposes (Garty, 2001). This property of lichens not only provides an efficient strategy to monitor the integrity of natural ecosystems, but also reinforces the prominent adaptability of lichens to unfavorable environmental conditions. The lichens utilized in this study resemble a consortium that can be found in various old-growth forests. Thus the changes in the community structure of the associated bacteria and their functioning allows general comparisons of the adaptions in the selected models. In contrast, lichens that are more adapted to the presence of pollutants can accumulate far higher concentrations of various metals (Garty, 2001). We hypothesize that such adaptations always involve the entire holobiont and that this applies across different lichen species. Here, the microbiota has either an active function in the detoxification or has to adapt to the adverse conditions like it was shown in the present study. Further studies will show if the observed adaption strategies of the holobiont to elevated arsenic concentrations are similar for other environmental pollutants.

## Author Contributions

TC, IA, MG, and GB designed the study. KF provided specific expertise related to qualitative and quantitative arsenic analyses. QV and TC conducted laboratory experiments required for the quantifications of arsenic. QV analyzed the samples under guidance of KF. TC, IA, and AE performed bioinformatic analyses with the metagenome datasets. TC evaluated the RT PCR-based quantifications. TC and AE subjected the RT PCR data to statistical analyses. TC, KF, MG, and GB wrote the manuscript. All authors read and approved the final version of the manuscript.

## Conflict of Interest Statement

The authors declare that the research was conducted in the absence of any commercial or financial relationships that could be construed as a potential conflict of interest.

## Footnotes

1 http://metagenomics.anl.gov

2 http://ncbi.nlm.nih.gov/genbank

3 http://ncbi.nlm.nih.gov
