# Supplementary material for: Adaptions of Lichen Microbiota Functioning Under Persistent Exposure to Arsenic Contamination
Source: Front Microbiol. 2018 Nov 30;9:2959. doi: 10.3389/fmicb.2018.02959 (PMC6283889; doi:10.3389/fmicb.2018.02959)
Supplement: Supplementary file 1 [file Data_Sheet_1.docx]

**Supplementary Data**

**Adaptions of lichen microbiota functioning under persistent exposure to arsenic contamination**

**Tomislav Cernava^1#^, Qerimane Vasfiu^2^, Armin Erlacher^1^, Ines Aline Aschenbrenner^1^, Kevin Francesconi^2^, Martin Grube^3^, and Gabriele Berg^1^**

^1^Institute of Environmental Biotechnology, Graz University of Technology, Petersgasse 12, 8010 Graz, Austria

^2^Institute of Chemistry, NAWI Graz, University of Graz, Universitätsplatz 1, 8010 Graz, Austria

^3^Institute of Plant Sciences, Karl-Franzens-University, Holteigasse 6, 8010 Graz, Austria

**#Correspondence**:

Tomislav Cernava, Environmental Biotechnology, Graz University of Technology, Petersgasse 12/I, 8010 Graz, Austria.

e-mail: tomislav.cernava@tugraz.at

telephone: +43 316 873 8423, fax: +43 316 873 8819

**Running title:** Arsenic-induced changes in the lichen microbiome

**Submitted to:** Frontiers in Microbiology

**Research topic:** The Microbial Ecology of Metalliferous Mine Waste Reclamation

**Research article**

**Key words:** arsenic pollution, lichen, lichen microbiome, arsenic resistance, holobiont

***Standards and instrumentation for the identification and quantification of arsenic species in lichen samples***

Water (18.2 MΩ cm) purified with a Millipore system (Millipore GmbH, Vienna, Austria) was used for all analytical procedures. Malonic acid (>99%) was obtained from Sigma Aldrich (Vienna, Austria), and nitric acid (>65%, p.a.), aqueous ammonia 25% (p.a.), hydrogen peroxide (30%, p.a.) and trifluoroacetic acid (>99,9%, p.a.) were obtained from Carl Roth GmbH (Karlsruhe, Germany). For the determination of arsenic species by HPLC/ICPMS, standard solutions were prepared for arsenate (As(V)), from Na_2_HAsO_4_. 7H_2_O and arsenite (As(III)), from NaAsO_2_, both from Merck Darmstadt, Germany, and dimethylarsinate (DMA), prepared from sodium dimethylarsinate purchased from Fluka Chemie (Buchs, Switzerland); methylarsonate (MA) was prepared in-house from As_2_O_3_ and CH_3_I (Meyer reaction). In addition, the following standards were prepared from previously synthesized in-house arsenic compounds (purity >99% by NMR and HPLC/mass spectrometry); arsenobetaine (AB), trimethylarsine oxide (TMAO), arsenocholine (AC), and tetramethylarsonium ion (TETRA). Internal standards used were germanium and indium (Carl Roth GmbH, Karlsruhe, Germany). Certified reference materials were: Rice Flour ERM BC 211 IRMM (Institute for Reference Materials and Measurements, Geel, Belgium); San Joaquin soil NIST SRM 2709 (National Institute of Standards and Technology, Gaithersburg, Maryland, USA); and lichen IAEA 336 (International Atomic Energy Agency, Vienna, Austria).

Samples were sonicated by using a Sonoplus Ultrasonic Homogenizer HD 2200 (BANDELIN electronic GmbH, Berlin, Germany). Centrifugation was performed with a Hettich 2043 Mikroliter (Andreas Hettich GmbH & Co. KG, Tuttlingen, Germany). For acid digestion a Ultraclave III microwave system (MLS GmbH, Leutkirch, Germany) was used. HPLC was performed with an Agilent 1100 series system (Agilent Technologies, Waldbronn, Germany), which was equipped with a binary pump, a vacuum degasser, column oven, and an autosampler. ICPMS measurements were performed with an Agilent 7900 series instrument, which was equipped with a Burgener Ari Mist HP nebulizer (Burgener Research Inc, Mississauga, Canada) and a Scott double pass spray chamber.

**Table S1.** Total arsenic content (µg/kg, dry mass) in analyzed lichen and soil samples. Samples that were obtained in the contaminated region (Gasen, Austria; 47°23'04.1"N 15°34'30.1"E) are highlighted with an asterisk (*).

| Sample name | Total As (mg/kg) | |
| --- | --- | --- |
| *Lobaria* (Bad Aussee) SW Weg^1^ | 0.209 | ± 0.020 |
| *Lobaria* (Bad Aussee) SW Groß^1^ | 0.139 | ± 0.010 |
| *Lobaria* (Bad Aussee) SW Kastanie^1^ | 0.140 | ± 0.010 |
| *Lobaria* NaClO^1^ | 0.205 | ± 0.030 |
| *Lobaria* Johnsbach | 0.266 | ± 0.010 |
| *Ramalina^1^* | 0.151 | ± 0.010 |
| *Ramalina* NaClO^1^ | 0.329 | ± 0.010 |
| *Cetrelia^1^* | 0.080 | ± 0.008 |
| *Cetrelia* NaClO^1^ | 0.225 | ± 0.010 |
| *Peltigera* | 1.002 | ± 0.040 |
| *Cladonia** | 2.276 | ± 0.670 |
| *Pseudovernia** | 6.362 | ± 0.740 |
| *Platismatia** | 2.207 | ± 0.240 |
| *Usnea** | 0.722 | ± 0.070 |
| *Hypogymnia** | 1.747 | ± 0.130 |
| Acer bark | 0.025 | ± 0.003 |
| Johnsbach soil | 4.406 | ± 0.050 |
| Graz soil | 7.224 | ± 0.430 |
| Gasen soil* | 72.947 | ± 1.320 |

**^1^**These lichen samples were used only for general assesments of arsenic contents; they were not included in the deepening molecular analyses.


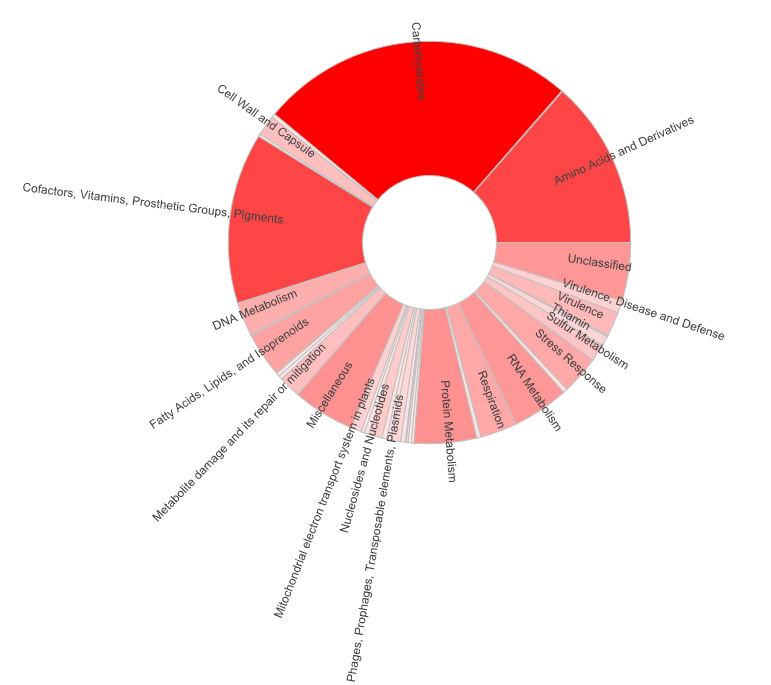


**Figure S1.** Functional profile of *Planctomycetacia* in the *C. furcata* metagenome. Reads assigned to *Planctomycetacia* were extracted from the metagenomic dataset and subjected to BLASTx searches against the NCBI-NR database (ncbi.nlm.nih.gov). All BLAST hits were hierarchically clustered with the SEED classification within MEGAN5 (Huson *et al*., 2011).


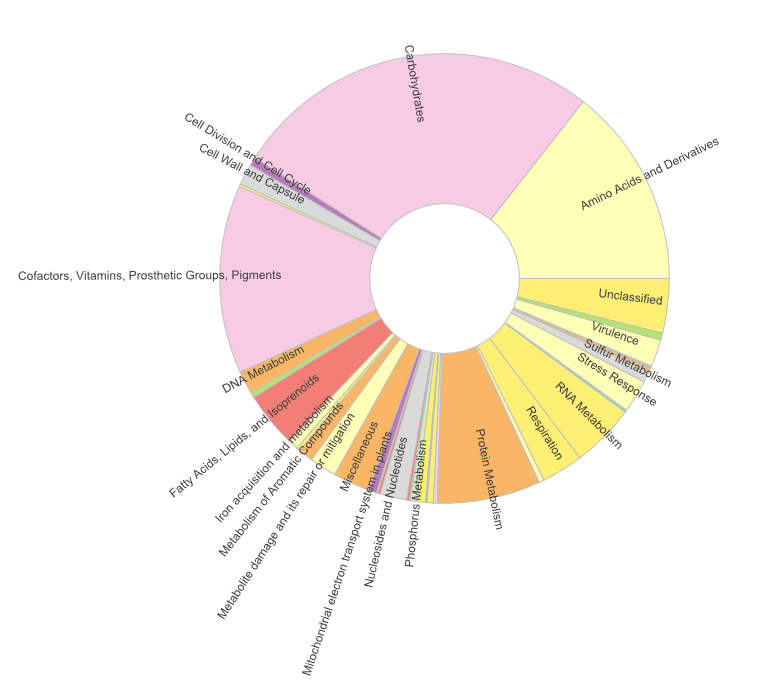


**Figure S2.** Functional profile of *Pedobacter* in the *C. furcata* metagenome. Reads assigned to *Pedobacter* were extracted from the metagenomic dataset and subjected to BLASTx searches against the NCBI-NR database (ncbi.nlm.nih.gov). All BLAST hits were hierarchically clustered with the SEED classification within MEGAN5 (Huson *et al*., 2011).

**
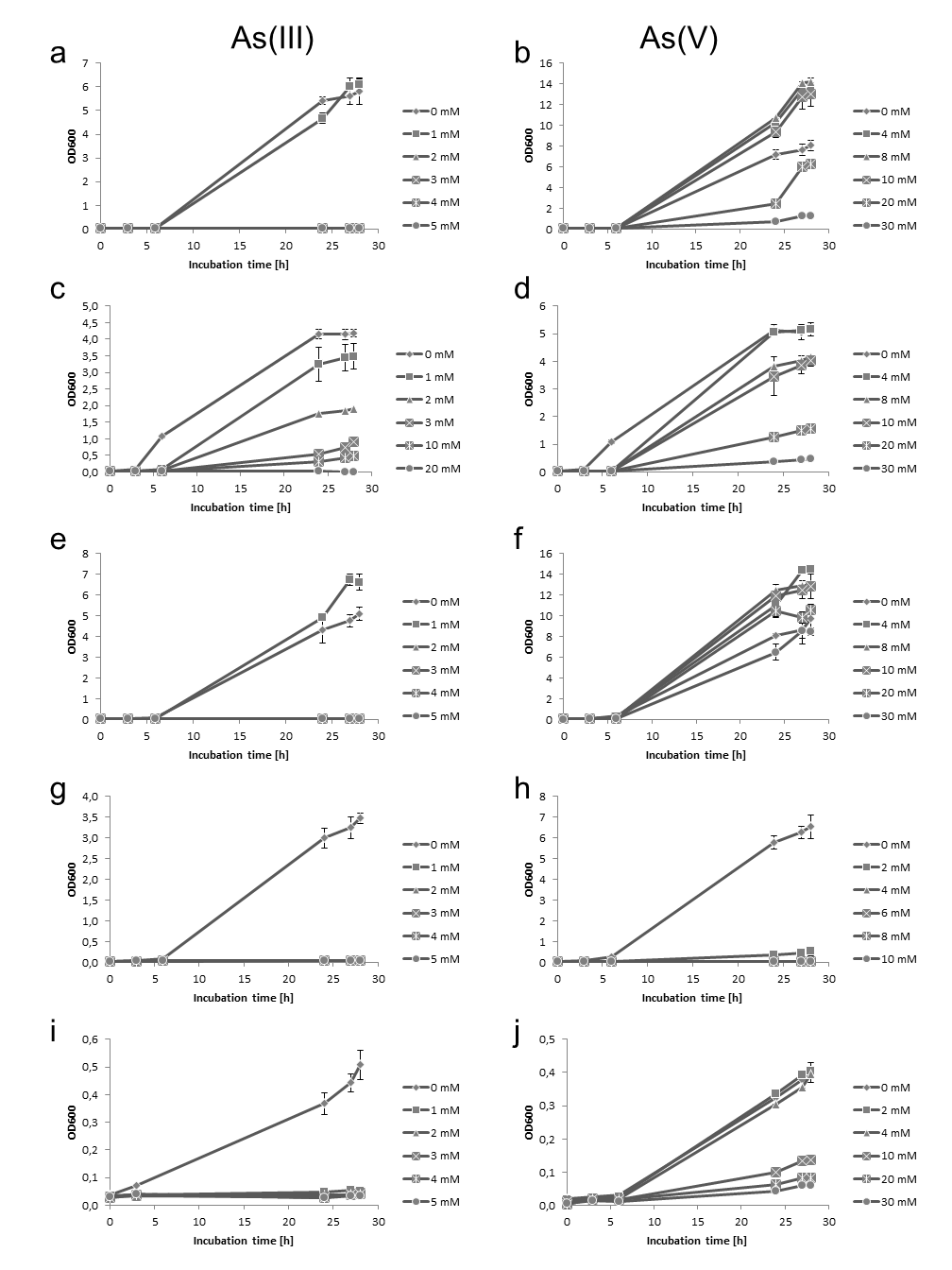
**

**Figure S3.** Isolated bacteria from three lichen species were grown in arsenic-supplemented media while their growth was monitored. *Micrococcus luteus* 29P4R (a, b)*; Staphylococcus warneri* 50P3R (c, d); *Micrococcus luteus* 77P3BRAB (e, f); *Pedobacter roseus* 127P3BR (g, h); *Leifsonia poae* 583P1B (i, j); growth curves were not obtained for *Streptomyces spiroverticillatus* 530P1B owing to agglomerate formation in the fluid media.
